# Non-invasive imaging of the coronary arteries

**DOI:** 10.1093/eurheartj/ehy670

**Published:** 2018-11-02

**Authors:** Philip D Adamson, David E Newby

**Affiliations:** 1 BHF Centre for Cardiovascular Science, University of Edinburgh, Room SU 305, Chancellor’s Building, 49 Little France Cres, Edinburgh, UK; 2 Christchurch Heart Institute, Department of Medicine, University of Otago, 2 Riccarton Ave, Christchurch, New Zealand

**Keywords:** Coronary heart disease, Computed tomography, Positron emission tomography, Magnetic resonance imaging

## Abstract

Non-invasive imaging of the coronary arteries is an enterprise in rapid development. From the research perspective, there is great demand for *in vivo* techniques that can reliably identify features of high-risk plaque that may offer insight into pathophysiological processes and act as surrogate indicators of response to therapeutic intervention. Meanwhile, there is clear clinical need for greater accuracy in diagnosis and prognostic stratification. Fortunately, ongoing technological improvements and emerging data from randomized clinical trials are helping make these elusive goals a reality. This review provides an update on the current status of non-invasive coronary imaging with computed tomography, magnetic resonance, and positron emission tomography with a focus on current clinical applications and future research directions.

## Introduction

Despite remarkable diagnostic and therapeutic advances in recent decades, coronary heart disease remains the largest single cause of death in Europe.^1^ In order to address this residual burden of morbidity and mortality, clinicians need greater access to imaging techniques that can improve the detection and prognostic classification of patients at risk of cardiovascular events.

Non-invasive imaging of the coronary arteries holds genuine promise that these hopes may be realized via both well-established diagnostic technologies, such as computed tomography coronary angiography (CTCA), and exciting new developments such as positron emission tomography (PET) and magnetic resonance coronary angiography (MRCA). Indeed, the results of large multicentre clinical trials are now emerging which will help clarify the optimal strategies for incorporating such tools into clinical practice.

To better understand the role and relative merits of the various options for non-invasive imaging, it is helpful to appreciate the processes of plaque biology and pathophysiology. Such background knowledge holds relevance to clinicians as it informs the rationale for imaging coronary atherosclerosis and the histological processes that we endeavour to identify through these investigations.

## Plaque biology as it relates to non-invasive imaging

**Figure 1:**
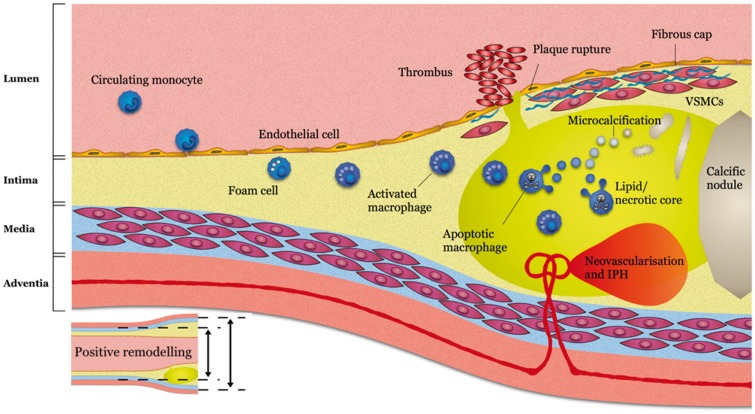
Imaging targets of high-risk plaque. Reused from Adamson *et al.*^2^ Circulating monocytes migrate into early intimal thickening where they phagocytose lipid becoming foam cells and activated macrophages detectable on ^68^Ga-DOTATATE positron emission tomography. Vascular remodelling can be detected on computed tomography coronary angiography prior to luminal stenosis developing. As the lipid core develops this can be detected as low-density signal on computed tomography coronary angiography. The resulting hypoxic environment prompts neovascularization with friable vessels prone to intraplaque haemorrhage, both of which can be detected on magnetic resonance coronary angiography. A necrotic core develops with microvesicles arising from apoptotic macrophages and vascular smooth muscle cells giving rise to microcalcifications detectable on ^18^F-fluoride positron emission tomography before coalescing into more stable calcific nodules detectable on computed tomography calcium scans. Rupture of the fibrous cap may result in intraluminal thrombosis detectable on magnetic resonance coronary angiography.

Atherosclerosis is a chronic inflammatory disease characterized by the formation of lipid-rich plaques. Intimal thickening is a near universal development by early adulthood and begins with an increase in vascular smooth muscle cells within the subintimal space. This is associated with concurrent insudation of circulating lipoproteins across the vascular endothelium where they are bound by an extracellular proteoglycan rich matrix. Oxidation of these lipoproteins initiates an inflammatory cascade as endothelial and smooth muscle cells express cellular adhesion molecules that promote migration and differentiation of circulating monocytes. The resultant macrophages, particularly the M1-subtype, act to promote a persistent maladaptive response leading to the development of the archetypal high-risk plaque; the thin-cap fibroatheroma. The hallmarks associated with high-risk plaque can be loosely categorized as those either related to the macroscopic structure of the plaque or to the biological processes occurring within it. Indeed, histological and imaging data have consistently demonstrated that culprit plaques responsible for myocardial infarction have the following characteristics: a large plaque volume, a lipid-rich necrotic core, positive remodelling, peripheral neovascularization, micro-calcification, intra-plaque haemorrhage, chronic inflammation, and a thin fibrous cap. Each of these characteristics represents a potential imaging target for *in vivo* identification of high-risk plaques and for guiding subsequent therapeutic modification (*Figure 1*).^2^

## Aims of non-invasive coronary imaging

**Figure 2:**
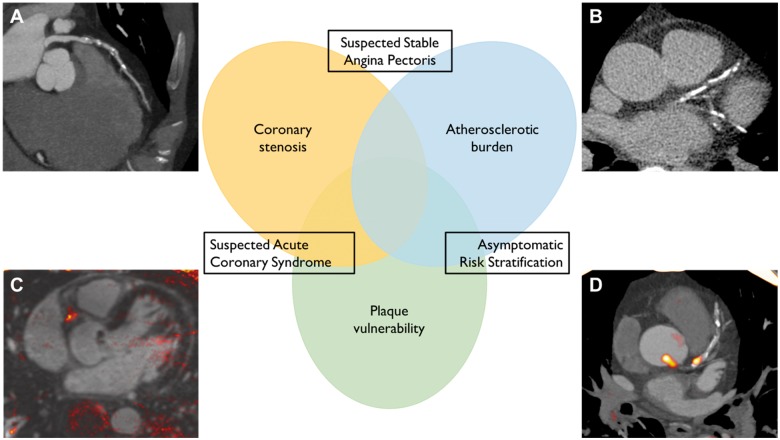
Complementary roles of non-invasive coronary imaging. The use of non-invasive coronary imaging can be considered in three contexts, each with specific objectives that may inform the choice of imaging modality. (*A*) Computed tomography angiography provides accurate assessment of coronary stenosis that can guide management of patients with suspected stable angina and rule out Type 1 myocardial infarction in patients with potential acute coronary syndrome. (*B*) Coronary artery calcium scanning is able to reliably quantify overall atherosclerotic burden and improve risk stratification in asymptomatic individuals. (*C*) T_1_-weighted magnetic resonance coronary angiography can identify features of atherosclerotic instability including intracoronary thrombus and intraplaque haemorrhage and may be of value in suspected acute coronary syndrome or asymptomatic risk stratification. (*D*) Positron emission tomography can employ specific tracers designed to target markers of plaque vulnerability that may improve prognostic assessment or act as a surrogate of therapeutic response in asymptomatic patients.

In evaluating the clinical role of non-invasive imaging, it is important to distinguish between patients based on the presence or absence of symptoms likely to be related to myocardial ischaemia. The former group comprises individuals with suspected stable angina pectoris or possible acute coronary syndromes. Here, the diagnostic objective is to identify or to exclude the presence of obstructive plaque causing sufficient luminal compromise that myocardial blood flow may be insufficient to meet metabolic demand. In contrast, coronary imaging in the asymptomatic population is largely targeted at estimating the risk of future events through the identification of atherosclerotic burden including non-obstructive disease and high-risk plaque (*Figure 2*). This review focusses on non-invasive imaging of coronary plaque structure and pathophysiology and will not describe the additional utility of these modalities in the assessment of coronary flow and myocardial ischaemia.

The four most developed non-invasive coronary imaging modalities include computed tomography coronary artery calcium (CAC) scoring, CTCA, MRCA, and positron PET—usually employed in combination with either computed tomography or magnetic resonance imaging (*Table 1*).

**Table 1:** Comparison of non-invasive coronary imaging modalities

Imaging parameters	CT calcium scan	CT coronary angiography	MRCA	PET
Image acquisition				
Scan duration	0.5–10 s	0.5–10 s	10–20 min	60–90 min (tracer uptake)15–30 min/PET bed
Spatial resolution	1.5–3.0 mm	0.5–1.0 mm	1.0–2.0 mm	4.0–10.0 mm (tracer dependent)
Temporal resolution	240–420 ms	240–420 ms (65 ms with dual-source CT)	<60 ms	Minutes
Radiation exposure	<1 mSv	1–10 mSv (protocol dependent)	Nil	6–15 mSv (less in PET-MR)
Advantages	Wide availability Low cost Low radiation exposure Large evidence base to support prognostic implications	Short scan time Wide availability Best spatial resolution Robust evidence to support use	Radiation free imaging Allows concurrent assessment of cardiac function Not limited by coronary calcification Soft tissue characterization	Tracers can be developed to target almost any structural or pathophysiological process of interest
Limitations	Limited spatial resolution Non-calcified (potentially high-risk) plaque not detectable	Requires adequate heart rate control Risk of contrast reaction/nephropathy Imaging limited by dense coronary calcification and stents Radiation exposure	Limited spatial resolution Prolonged scan duration High cost Limited availability Claustrophobia Metallic implants	Poor spatial resolution Prolonged tracer uptake time and long scan duration Relatively high radiation exposure (PET-CT) although this can be substantially reduced with PET-MR High cost Very limited availability
Indications	Risk stratification in primary prevention for individuals at low-intermediate risk of cardiovascular events	Non-invasive assessment of suspected stable angina in patients with intermediate pre-test probability of coronary artery disease	Anomalous coronary arteries Follow-up of Kawasaki disease (coronary aneurysms) Assessment of coronary bypass grafts	Research purposes only at present

CT, computed tomography; MRCA, magnetic resonance coronary angiography; PET, positron-emission tomography.

## Coronary artery calcium scoring

Atherosclerotic calcification is a well-described process, in part occurring as a healing response to pathological inflammation within the plaque. In its earliest stages, extracellular debris acts as a nidus for calcium deposition and the resultant microcalcifications have been shown to increase the likelihood of rupture of the surface of fibroatheromas. In more advanced disease—by which stage calcium is detectable on computed tomography—these microcalcifications coalesce into large, calcific nodules.

The assessment of coronary artery calcification, is one of the most enduring applications for non-invasive coronary artery imaging. The relationship between coronary calcification and obstructive coronary artery disease was initially determined from chest radiography and confirmed with findings from *in vivo* invasive coronary angiography and *ex vivo* histology.^3^^,^^4^ The subsequent introduction of electron-beam computed tomography substantially improved diagnostic sensitivity, and scans are now commonly performed on non-contrast images obtained from multi-detector computed tomography scanners at sub-millisevert radiation doses.^5^ The CAC scan consists of a non-contrast, gated computed tomography of the heart acquired during a short period of held inspiration. Arterial calcium is defined as the presence of a lesion with a density >130 Hounsfield units across an area of at least 1 mm^2^. Atherosclerotic calcification can be reported by volume or mass, but most commonly is described in Agatston units (AU), a semi-quantitative measure that incorporates aspects of calcium density and distribution. Coronary calcium scoring has been evaluated both in patients with suspected angina and asymptomatic populations.

Uncertainty persists regarding the utility of CAC scoring amongst symptomatic individuals. In these patients, a CAC score of >0 has a diagnostic sensitivity for identifying a coronary stenosis of ≥50% of between 0.89 and 0.99.^6–8^ However, the corresponding specificity is poor, ranging from 0.40 to 0.59. Consequently, in low-risk populations, such as patients with atypical symptoms in the outpatient clinic, the low positive predictive value will necessitate additional diagnostic imaging in many cases. Conversely, in situations with high pre-test probability of disease, for example, amongst troponin positive patients in the Emergency Department, the test will have an unacceptably high ‘false-negative’ rate. This is a particular concern as non-calcified plaques often exhibit additional high-risk features on alternative imaging modalities and are therefore more likely to rupture with resultant myocardial infarction. Consequently, CAC scoring is not recommended in the diagnostic assessment of symptomatic chest pain patients.

Accepting that not all plaques contain calcium detectable on computed tomography, the total CAC score has been demonstrated in histopathological and intravascular ultrasound studies to offer an acceptable approximation of the overall plaque burden for an individual. Given the association between CAC and both plaque burden and coronary obstruction, it is perhaps unsurprising that numerous reports have confirmed the prognostic value of such scores. The risk stratification provided is in addition to established clinical and biochemical risk factors, and the St Francis Heart Study found an improvement in the *c*-statistic for clinical events from 0.69 to 0.79 when added to the Framingham risk score^9^; a finding that has been confirmed in several larger cohort studies including the MESA (Multi-Ethnic Study of Atherosclerosis),^10^ and HNR (Heinz Nixdorf Recall—Risk Factors, Evaluation of Coronary Calcium and Lifestyle)^11^ trials. This improvement is of most value in intermediate risk patients without established cardiovascular disease who may be considering whether to commence primary prevention therapies. In this context, both the 2013 ACC/AHA and 2016 ESC guidelines on the prevention of cardiovascular disease give a Class IIb (may be considered) recommendation to CACS.^12^^,^^13^

## Computed tomography coronary angiography

The clinical application of CTCA was long delayed by the problems of cardiac motion and high radiation exposure. Fortunately, advances in scanner technology and the introduction of prospective electrocardiographic (ECG)-gating have largely overcome these challenges and diagnostic image quality can now be obtained in 95% of scans in unselected populations.^14^ Where image quality remains poor, it is commonly related to motion artefacts at high heart rates, dense coronary calcification, or coronary stents. Such limitations can be minimized through appropriate patient selection and preparation, including judicious use of beta-blockers as part of the scanning protocol. Clinical imaging is performed on ≥64-slice computed tomography scanners, using intravenously administered contrast media and can be performed with radiation exposures in the range of 3–5 mSv.

### Computed tomography coronary angiography as a diagnostic tool for coronary obstruction

One of the earliest proposed uses for CTCA related to improving the selection of patients presenting with suspected stable angina who required invasive coronary angiography and initial studies on this imaging modality focused on diagnostic accuracy with regards to detecting or excluding obstructive CAD. A meta-analysis published in 2007 described a sensitivity of 93% and specificity of 96% on a per-segment basis for the detection of coronary stenoses >50%,^15^ and in symptomatic patients with an intermediate pre-test probability of obstructive coronary artery disease, the negative predictive value of a negative CTCA is reported to be >95%.^16^ Based on the results of these diagnostic accuracy studies, CTCA has gained endorsement by international guidelines as a reasonable choice of non-invasive test in appropriately selected patients.^17^^,^^18^

### Computed tomography coronary angiography for prognosis

**Figure 3:**
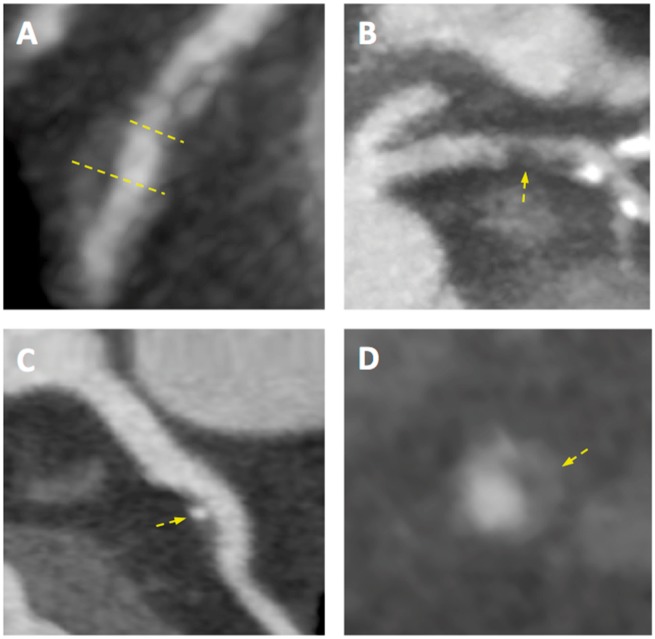
Adverse coronary plaque characteristics identified on computed tomography coronary angiography. Coronary atherosclerotic plaque features detected using computed tomography coronary angiography including (*A*) positive remodelling—defined as an outer vessel diameter (large yellow line) 10% greater than the mean diameter of the segments immediately proximal (small yellow line) and distal to the plaque; (*B*) low attenuation plaque—defined as a focal central area of plaque with an attenuation density of <30 Hounsfield Units (yellow arrow); (*C*) spotty calcification—defined as focal calcification within the coronary artery wall <3 mm in maximum diameter (yellow arrow); and (*D*) the ‘napkin ring’ sign—defined as a central area of low attenuation plaque with a peripheral rim of high attenuation (yellow arrow).

In addition to diagnosing coronary obstruction, the ability of CTCA to accurately quantify the anatomical distribution and severity of coronary atherosclerosis enables it to provide valuable prognostic information. As for invasive imaging, there is a clear stepwise worsening of prognosis associated with increasing numbers of diseased vessels.^19^ Indeed, due to its sensitivity in the detection of non-obstructive coronary atheroma and ability to identify additional adverse plaque characteristics, risk stratification is a particular strength of CTCA. As previously mentioned, histopathological and invasive coronary imaging studies have identified a number of features that are commonly present in plaques at risk of coronary rupture.^20^ Correlates of these features have been described for non-invasive imaging with CTCA and include the presence of positive remodelling, low attenuation plaque, spotty calcification, and the ‘napkin ring’ sign (*Figure 3*).^21^ More recently, changes in the composition of perivascular adipose tissue have also been described that are detectable on CTCA, correlate with histological evidence of plaque inflammation, and may arise in response to paracrine signalling.^22^ Numerous studies have reported an association between such adverse plaque characteristics and an increased risk of subsequent cardiovascular events.^19^^,^^21^^,^^23^^,^^24^ Furthermore, as for quantification of coronary calcification, a variety of prognostic scores have been described. At the simplest level, the segment involvement score (SIS) sums the number of diseased coronary segments, whilst the stenosis severity score (SSS) also incorporates a weighting factor for stenotic severity.^19^ More recently, the computed tomography-adapted Leaman score, combining stenotic severity, myocardium at risk, and high-risk plaque features, appears to improve risk stratification further.^25^

### Randomized controlled trials testing computed tomography coronary angiography

In contrast with most radiological investigations used in medical practice, the clinical utility of CTCA has been rigorously determined in a series of randomized clinical trials. In a comparison with exercise ECG testing, the CAPP (Cardiac CT for the Assessment of Pain and Plaque) trial (*n* = 500) demonstrated an improvement in angina-related quality of life, with the use of CTCA.^26^ This trial was underpowered for hard clinical events but identified a corresponding reduction in unplanned hospital admissions amongst those in the CTCA intervention arm. More recently, the much larger PROMISE (Prospective Multicenter Imaging Study for Evaluation of Chest Pain) and SCOT-HEART (Scottish Computed Tomography of the HEART) trials have reported their findings. PROMISE (*n* = 10 003) randomized intermediate-risk symptomatic patients being evaluated for the presence of coronary heart disease to CTCA or non-invasive functional testing (67% nuclear stress imaging, 23% stress echocardiography, 10% exercise ECG).^27^ The median duration of follow-up was 25 months and no difference in the primary composite endpoint (death, myocardial infarction, hospitalization for unstable angina, or major procedural complication) was demonstrated despite a downstream reduction in the rate of unnecessary invasive coronary angiograms and apparent reductions in death or myocardial infarction at 12 months. The SCOT-HEART trial (*n* = 4146) investigated the utility of adding CTCA to standard care (predominantly exercise ECG) in a broad population of patients seen in rapid access chest pain clinics across Scotland.^14^ The primary endpoint of diagnostic certainty at 6-weeks was increased with CTCA. Recently, the 5-year composite clinical outcome of coronary death or non-fatal myocardial infarction has been reported, with a marked 40% relative risk reduction in the CTCA arm of the trial.^28^ In aggregate, these trials provide powerful evidence of benefit for a CT first approach in the assessment of stable chest pain.^29^

Randomized trials of CTCA have also been conducted in the Emergency Department setting amongst patients with suspected acute coronary syndromes. Examples include the CT-STAT (Coronary Computed Tomographic Angiography for Systematic Triage of Acute Chest Pain Patients to Treatment, *n* = 1370), ROMICAT-II (Rule Out Myocardial Infarction/ischaemia Using Computer Assisted Tomography, *n* = 1000), and ACRIN-PA (CT Angiography for Safe Discharge of Patients with Possible Acute Coronary Syndromes, *n* = 699) trials.^30–32^ Compared with standard care, CTCA reduced both the time required to establish a diagnosis and the overall length of stay, albeit with no difference in hard clinical outcomes. However, these trials were performed prior to the introduction of high-sensitivity cardiac troponin assays, which allow more rapid rule-out of myocardial infarction in the Emergency Department setting.^33^^,^^34^ The more contemporary BEACON (Better Evaluation of Acute Chest Pain with Coronary Computed Tomography Angiography, *n* = 500) trial employed such assays and perhaps consequently was unable to demonstrate any benefit regarding length of stay with the use of CTCA.^35^ This finding has been corroborated by a *post hoc* analysis of the ROMICAT I and II trials where stored samples were used for the measurement of high-sensitivity cardiac troponin I.^36^

Less evidence exists to inform the role of CTCA in the management of asymptomatic patients at risk of cardiovascular disease, and such an approach has been investigated in only a single trial to date. The FACTOR-64 (Screening For Asymptomatic Obstructive Coronary Artery Disease Among High-Risk Diabetic Patients Using CT Angiography, Following Core 64) trial randomized 900 patients with established diabetes mellitus but no prior history of cardiovascular disease to standard care or CTCA.^37^ Intensive treatment of co-morbid vascular risk factors was strongly encouraged in those identified with coronary atheroma on non-invasive imaging. Due to excellent use of preventative therapies in the standard care arm, CTCA was associated with only a small incremental reduction in cholesterol profiles and event rates were low in both treatment groups; less than one quarter of that expected. Overall, despite a numerical trend, no improvement in the primary composite cardiovascular endpoint was identified (hazard ratio 0.69, 95% confidence interval 0.41–1.16; *P* = 0.16).

### Future research directions for computed tomography coronary angiography

Having demonstrated the benefits of CTCA in the diagnosis of suspected stable angina, further work is underway to test further roles for this technique in clinical practice. The RAPID-CTCA (Rapid Assessment of Potential Ischaemic Heart Disease with CTCA) trial is a multicentre study recruiting patients with suspected acute coronary syndrome and an additional risk factor; such as positive cardiac troponin, ischaemic changes on the ECG, or an established history of CAD. Patients will be randomized to early CTCA or standard care—in many cases likely to include invasive coronary angiography. The trial is powered with regards to the primary endpoint of all-cause death or recurrent non-fatal myocardial infarction at 1-year and plans to recruit 2500 patients.^38^ Another ongoing study of CTCA in the acute coronary syndrome setting is the TARGET-CTCA (Troponin in Acute chest pain to Risk stratify and Guide EffecTive use of Computed Tomography Coronary Angiography) trial. This study exploits the potential of high-sensitivity troponin to identify an at risk subgroup of the suspected ACS population where the peak troponin concentration is mildly increased but remains below the 99th centile upper reference limit. The trial is based on the premise that many of these patients are currently discharged without a definitive aetiology for their symptoms being established and that CTCA, by providing diagnostic clarification, may allow better therapeutic targeting.

## Magnetic resonance coronary angiography

**Figure 4:**
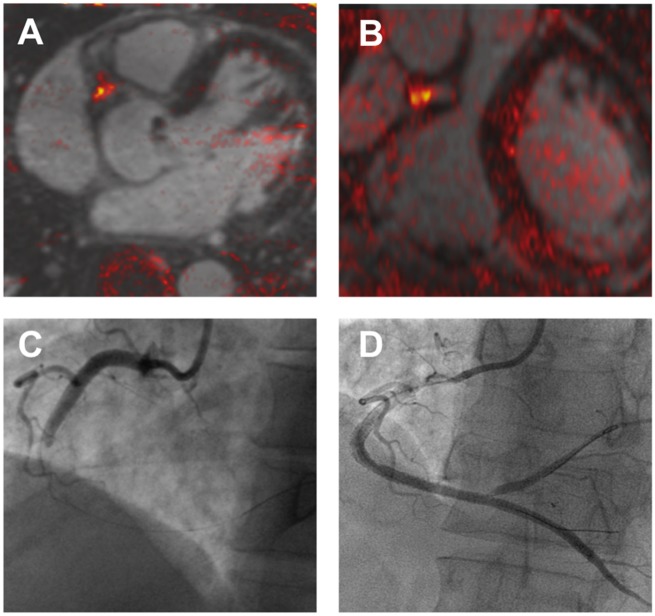
Coronary atherosclerosis T_1_-weighted characterization with integrated anatomical reference (CATCH). T_1_-weighted magnetic resonance coronary angiogram of a patient who presented with an inferior myocardial infarction shows evidence of a focal high intensity lesion (arrows) in the right coronary artery on magnetic resonance imaging (*A* and *B*). Subsequent coronary angiogram demonstrated occlusion of the mid-right coronary artery (*C*) with restoration of flow following thrombus aspiration (*D*).

Magnetic resonance angiography is a widely accepted technique for imaging larger conduit vessels, particularly the carotid arteries and abdominal aorta. However, due to limitations related to spatial resolution and long-scan times, coronary imaging with MRCA currently has limited indications in clinical practice, except in the assessment of anomalous coronary arteries, coronary aneurysms, and coronary bypass grafts. Nevertheless, MRCA has important potential strengths that have maintained ongoing research interest. These include the ability for luminal visualization in the presence of dense calcification, the absence of ionizing radiation, the possibility for concomitant functional imaging of the myocardium, and the potential for detailed tissue characterization. A recently described example of the latter, is the identification of high-intensity plaque on non-contrast T_1_-weighted imaging (*Figure 4*).^39^ T_1_-weighted imaging targets methaemoglobin, a component of thrombus and intraplaque haemorrhage, and high-intensity plaque appears to be an MRCA analogue for low-density plaque on CTCA that is associated with high-risk features on invasive imaging with intravascular ultrasound or optical coherence tomography.^40^^,^^41^ The presence of high-intensity plaque also correlates with increased risk of procedure-related myocardial injury during percutaneous coronary intervention.^39^

Stepwise advances in imaging, moving from single-slice breath-hold sequences, through free-breathing whole-heart scanning, and the introduction of 3 Tesla magnets have brought a broader clinical role for MRCA closer. Nevertheless, in the assessment of suspected stable angina, MRCA currently has only modest diagnostic accuracy, with a recent meta-analysis of 24 studies reporting a pooled sensitivity and specificity for the detection of >50% stenosis on invasive coronary angiography of 89% and 72%, respectively.^42^ Whilst diagnostic performance can be increased with the use of gadolinium-based intravascular contrast agents, MRCA undoubtedly remains in its infancy.

## Positron emission tomography

Positron emission tomography is a non-invasive technique that is underpinned by molecularly targeted probes, conjugated to a radioactive isotope that undergoes beta decay. The probes vary widely in regards to structural complexity but each is chosen in order to bind with very high sensitivity to important components of a specific pathophysiological process of interest. After binding, the emitted positron travels a short distance *in vivo* before interacting with an electron. The resultant annihilation releases two photons in opposing directions which exit the body and are identified as coincident events by the encircling detector ring.^43^ Even more so than with magnetic resonance imaging, PET imaging of the coronary arteries has traditionally been challenged by the problems of prolonged scan times, spatial resolution and limited availability.^44^ Hybrid PET-CT and PET-MR scanners have begun to address these challenges and coronary PET imaging now appears to be a viable proposition. Although the scope to manufacture probes for molecular imaging targets is near-limitless, to date the majority of clinical research has related to three tracers of interest (*Table 2*).

**Table 2:** PET radiotracers for coronary atherosclerosis

Target	Ligand	Radiotracer	Application to date	Selected ongoing clinical trials
Macrophage activation	GLUT (1 and 3) and conversion by hexokinase to ^18^F-FDG-6-phosphate	^18^F-FDG	Prospective *in vivo* studies in extracardiac atherosclerosis Myocardial suppression required to evaluate coronary arteries	Vascular Inflammation in Psoriasis (NCT02187172, NCT03082729)
	Somatostatin receptor subtype 2	^68^Ga-DOTATATE	Prospective *in vivo* studies in cardiac and extracardiac atherosclerosis Retrospective *in vivo* studies in coronary artery disease	
	Translocator protein 18-kDa	^11^C-PK11195	Prospective *in vivo* study in carotid stenosis	
	Translocator protein 18-kDa	^11^C-PBR28	Clinical studies in healthy controls and multiple sclerosis	Cardiac Sarcoidosis (NCT02017522)
	Mannose receptor	^18^F-FDM	Preclinical cell culture model	
	Choline kinase phosphorylated to Phosphatidylcholine	^18^F-choline	Preclinical murine model	ESCAPPE (NCT02640313)
Apoptosis	Phosphatidylserine	^68^Ga-Annexin A5	Preclinical murine model	
Hypoxia	Reduction to amine derivative in low O_2_ environment	^18^F-FMISO	Preclinical murine model	
	Reduction to amine derivative in low O_2_ environment	^18^F-HX4	Proof of concept in carotid atherosclerosis	
Microcalcification	Hydroxyapatite	^18^F-fluoride	Prospective *in vivo* studies in coronary and extracardiac atherosclerosis	PREFFIR Study (NCT02278211) ROPPET-NAF (NCT03233243) PET-MR Imaging In Patients With Cardiac Amyloidosis (NCT03626584) Pilot Study-Magnetic Resonance Imaging for Global Atherosclerosis Risk Assessment (NCT02265250)
Angiogenesis	αVβ3 and αVβ5 integrin	^18^F-Fluciclatide	Prospective *in vivo* studies in cardiac and extracardiac atherosclerosis	
	αVβ3 integrin	^18^F-RGD-K5	*Ex vivo* human carotid studies	Carotid plaque imaging study NCT01968226

Adapted from Moss *et al.*^45^

### ^18^F-Fluorodeoxyglucose

^18^F-Fluorodeoxyglucose (^18^F-FDG), a non-specific marker of cellular inflammation was the first tracer to be investigated for coronary imaging with preclinical models showing a correlation between tracer uptake and increased macrophage activity.^46^ When used *in vivo* to image the carotid arteries, ^18^F-FDG uptake correlates with high-risk plaque features on CT and histological specimens. Carotid uptake has also been demonstrated to identify a reduction in atherosclerotic inflammation in response to treatment with simvastatin.^47^ Similar associations within the coronary vasculature are likely, but imaging in this location is made challenging by the intense myocardial uptake, often overwhelming the coronary signal.^48^

### ^18^F-Fluoride

**Figure 5:**
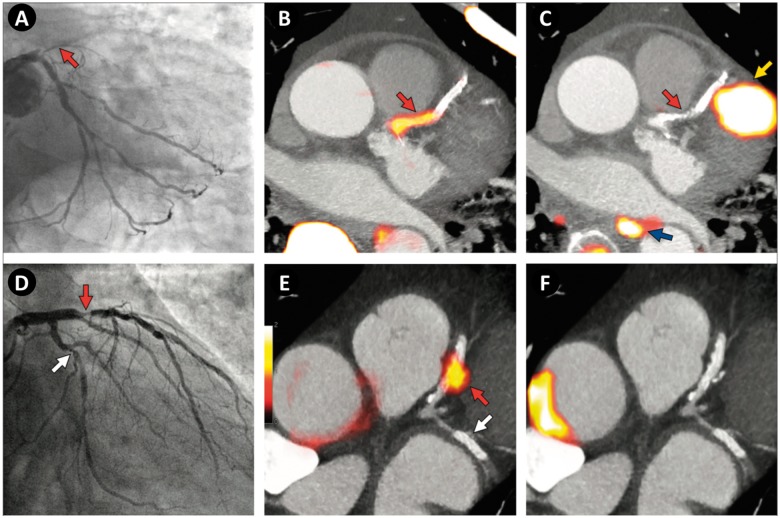
Focal ^18^F-fluoride and ^18^F-fluorodeoxyglucose uptake in patients with myocardial infarction and stable angina. (Top row, *A*–*C*) Patient with acute ST-segment elevation myocardial infarction with (*A*) proximal occlusion (red arrow) of the left anterior descending artery on invasive coronary angiography and (*B*) intense focal ^18^F-fluoride uptake (yellow-red) at the site of the culprit plaque (red arrow) on the combined positron emission and computed tomography coronary angiography (PET-CTCA). Corresponding ^18^F-fluorodeoxyglucose PET-CT image (*C*) showing no uptake at the site of the culprit plaque. Note the significant myocardial uptake overlapping with the coronary artery (yellow arrow) and uptake within the oesophagus (blue arrow). (Bottom row) Patient with anterior non-ST-segment elevation myocardial infarction with (*D*) culprit (red arrow; left anterior descending artery) and bystander non-culprit (white arrow; circumflex artery) lesions on invasive coronary angiography that were both stented during the index admission. Only the culprit lesion had increased ^18^F-NaF uptake on PET-CT (*E*) after percutaneous coronary intervention. Corresponding ^18^F-fluorodeoxyglucose PET-CT (*F*) showing no uptake either at the culprit or the bystander stented lesion. Note intense uptake within the ascending aorta. Adapted from Joshi *et al.*^47^

^18^F-Fluoride binds with high affinity to the exposed surface of hydroxyapatite, a key mineral component of vascular calcification. Initially developed for the detection of bony metastases, it is now recognized to enable the detection of early microscopic atherosclerotic calcification prior to the development of macroscopic calcification on CT imaging.^49^ In this context, tracer binding demonstrates intense signal in areas of active mineralization where large numbers of microcalcific deposits are present throughout the plaque, increasing strain on the fibrous cap, thereby potentially provoking plaque rupture. In contrast to ^18^F-FDG, ^18^F-fluoride is not limited by myocardial uptake and identifies the culprit artery in patients diagnosed with acute myocardial infarction (*Figure 5*).^48^ The ability of coronary imaging with ^18^F-fluoride PET-CT to improve risk stratification following myocardial infarction is currently being investigated in a prospective multicentre trial (NCT02278211).

### ^68^Ga-DOTATATE

^68^Gallium-labelled DOTATATE binds to the somatostatin receptor subtype 2 (SSTR2) found on the surface of macrophages, particularly the proinflammatory M1 subtype. Preclinical studies suggest it may be a superior marker of coronary macrophage activity than ^18^F-FDG and importantly myocardial uptake is minimal. A recent report demonstrates increased ^68^Ga-DOTATATE uptake in culprit coronary and carotid plaques and correlation with CT and histological evidence of high-risk plaque.^50^ Whether this information can be used to inform patient management remains to be determined.

## Conclusions

Non-invasive imaging of the coronary arteries is an enterprise in rapid development. From the research perspective, there is great demand for *in vivo* techniques that can reliably identify features of high-risk plaque that may offer insight into pathophysiological processes and act as surrogate indicators of response to therapeutic intervention. Meanwhile, there is clear clinical need for greater accuracy in symptom diagnosis and prognostic stratification. Fortunately, constant incremental enhancements in scanner technology and image post-processing are helping make these elusive goals a reality. To date, computed tomography, remains the most clinically applicable technique due to its broad availability and the strength of its evidence base. Coronary calcium scoring appears to be a useful technique for improving risk assessment in primary prevention, whilst CTCA is a valuable diagnostic test that improves diagnostic certainty and optimizes downstream management in symptomatic patients. At present, MRCA and PET remain largely investigational imaging modalities but landmark trials are now underway that will inform their future clinical role.
